# Plant Microbiomes: Do Different Preservation Approaches and Primer Sets Alter Our Capacity to Assess Microbial Diversity and Community Composition?

**DOI:** 10.3389/fpls.2020.00993

**Published:** 2020-07-03

**Authors:** Zhiguang Qiu, Juntao Wang, Manuel Delgado-Baquerizo, Pankaj Trivedi, Eleonora Egidi, Yi-Min Chen, Haiyang Zhang, Brajesh K. Singh

**Affiliations:** ^1^ Hawkesbury Institute for the Environment, Western Sydney University, Penrith, NSW, Australia; ^2^ Departamento de Sistemas Físicos, Químicos y Naturales, Universidad Pablo de Olavide, Seville, Spain; ^3^ Microbiome Cluster and Department of Agricultural Biology, Colorado State University, Fort Collins, CO, United States; ^4^ Global Centre for Land-Based Innovation, Western Sydney University, Penrith, NSW, Australia

**Keywords:** plant microbiome, sampling, preservation methods, amplicon sequencing, soil, sequencing primers

## Abstract

The microbial communities associated with plants (the plant microbiome) play critical roles in regulating plant health and productivity. Because of this, in recent years, there have been significant increase in studies targeting the plant microbiome. Amplicon sequencing is widely used to investigate the plant microbiome and to develop sustainable microbial agricultural tools. However, performing large microbiome surveys at the regional and global scales pose several logistic challenges. One of these challenges is related with the preservation of plant materials for sequencing aiming to maintain the integrity of the original diversity and community composition of the plant microbiome. Another significant challenge involves the existence of multiple primer sets used in amplicon sequencing that, especially for bacterial communities, hampers the comparability of datasets across studies. Here, we aimed to examine the effect of different preservation approaches (snap freezing, fresh and kept on ice, and air drying) on the bacterial and fungal diversity and community composition on plant leaves, stems and roots from seven plant species from contrasting functional groups (e.g. C3, C4, N-Fixers, etc.). Another major challenge comes when comparing plant to soil microbiomes, as different primers sets are often used for plant vs. soil microbiomes. Thus, we also investigated if widely used 16S rRNA primer set (779F/1193R) for plant microbiome studies provides comparable data to those often used for soil microbiomes (341F/805R) using 86 soil samples. We found that the community composition and diversity of bacteria or fungi were robust to contrasting preservation methods. The primer sets often used for plants provided similar results to those often used for soil studies suggesting that simultaneous studies on plant and soil microbiomes are possible. Our findings provide novel evidence that preservation approaches do not significantly impact plant microbiome data interpretation and primer differences do not impact the treatment effect, which has significant implication for future large-scale and global surveys of plant microbiomes.

## Introduction

The microbial communities associated with different plants compartments, from roots to leaves (the plant microbiome), play a crucial role in plant health and productivity (Köberl et al., 2013; Andreote et al., 2014; Berg et al., 2016; Rosier et al., 2016; Colla et al., 2017; Qiu et al., 2019). The plant microbiome can perform key functions in supplying nutrients and helping to control pathogens (Schmalenberger et al., 2008; Compant et al., 2010; Suárez-Moreno et al., 2012; Sessitsch and Mitter, 2015). Because of this, harnessing beneficial microbes associated with plants is considered a promising emerging tool to improve agricultural productivity and sustainability. Understanding the plant microbiome assembly mechanisms and how these microbiomes interact with their hosts is a fundamental first step to achieve this goal. Over the last few years, there has been an increasing number of studies using next generation sequencing to unveil the plant microbiome structure and dynamics (Berg et al., 2014; Agler et al., 2016; Hamonts et al., 2018). Particularly, amplicon sequencing has been widely used to discover the fundamental process of microbial assembly in plant germination, growth, metabolism and defence (Mayak et al., 2004; Weyens et al., 2009; Schmidt et al., 2014).

Despite the importance and potential opportunities offered by the plant microbiome, there are still multiple challenges that need to be addressed to advance our knowledge including lack of large-scale study to identify the processes that govern assembly and function of plant microbiomes. Large-scale studies in plant microbiomes is constraints by logistic issues including plant preservation after samples collection, and its important role in maintaining the original microbial community intact. For example, the snap freezing (in liquid nitrogen) preservation method for sampling and transporting to laboratory is considered the gold standard method for field surveys, as samples are immediately placed at −20°C or below after collection *in situ* to minimise the disruptions of the plant tissue and its microbiome (Agler et al., 2016; Timm et al., 2016; Hamonts et al., 2018). However, in some circumstances, snap freezing is impractical due to logistic and financial difficulties, especially when large number of samples are required from remote areas, or in global and regional studies. This has led to the development of alternative approaches for storing samples, which have also proven effective on non-plant samples, including the use of FTA cards (Song et al., 2016), ethanol (Estes et al., 2013; Koch et al., 2013), CTAB (Hammer et al., 2015) and RNAlater (Campbell et al., 2004; Sanders et al., 2014). However, most of these methods are not applicable for plant microbiome due to the requirement of the tissue integrity for downstream analysis. In plant microbiome studies, snap freezing is still the most common method for preserving plant materials (Agler et al., 2016; Deyett and Rolshausen, 2020), but in suboptimal conditions, air-dry with silica gel (Bazzicalupo et al., 2013) ice incubation or fridging (−4°C to 4°C, Kaushal et al., 2020) have also been used for sample preservation, but the effect of this approach on microbiome integrity has not been fully tested compared to snap freezing method. With the increasing interests of harnessing plant microbiome to sustainably promote crop productivity, more initiatives and projects aimed to unfold the plant microbiome from regional to global scales have been launched recently. Therefore, finding practical and cost-effective preservation approaches is critical to accommodate the ever-increasing number of samples for sequencing, and ultimately harnessing microbial-based knowledge for the development of sustainable agricultural technologies.

Another major challenge is associated with the fact that plant bacterial microbiomes are often assessed with different primer sets (e.g. 799F-1193R) than those used for soils (e.g. 341F-805R, 515F-806R). This is not an issue for fungi as plant and soil studies can sequence the same fungal ITS region without getting huge variance. In the case of bacteria, plant microbiome studies often use 16S rRNA gene primers (799F-1193R) targeting the V5–V7 region of the gene (Bai et al., 2015; Liu et al., 2017). Unlike for the primer sets most used for soils (e.g. 341F-805R; region V3–V4, Delgado-Baquerizo et al., 2016; Feng et al., 2016; 515F-806R; region V4, Caporaso et al., 2011; Caporaso et al., 2012; Walters et al., 2016), these plant microbiome primer sets minimise the sequencing of chloroplast and mitochondrial 16S rRNA gene (Beckers et al., 2016). Although alternative approaches are available such as using PNA blockers (Fitzpatrick et al., 2018), the efficiency to reduce the amplification of plant material was still far from ideal (Hamonts et al., 2018). This poses an important challenge, as the lack of demonstration that the primer set (799F-1193R) is valid for soils, and yields similar results to those from 341F-805R, limiting any attempt to compare both soil and plant microbiomes. Because of this, it is critical that we investigate whether the typical primer set used for plant microbiomes is also valid for soil, and provides comparable data to commonly used soil primer sets.

Here, we aim to 1) examine the effect of different preservation approaches on plant microbiome analysis and to identify the best preservation method to maintain sample integrity, and 2) to evaluate whether the plant primer pair targeting V5–V7 regions is valid for soil microbiomes, and provide similar results in this environment than those primers that target the V3–V4 region. To assess the effect of plant preservation methods, we implemented three preservation approaches commonly used in plant microbiome studies: a) silica gel desiccation, with samples incubated at room temperature until fully dehydrated; b) incubation on ice for 24 h; and c) snap freezing in liquid nitrogen immediately after sample collection and then transfer to −80°C. To further evaluate the variability of microbiome and distinct difference of the leaf traits that could potentially affect plant microbiome across species, we selected five plant species from contrasting functional groups, including C3 (wallaby grass *Austrodanthonia caespitosa*) and C4 (kangaroo grass *Themeda triandra* and rhodes *Chloris gayana*) grasses, the nitrogen fixing legume lucerne (*Medicago sativa*), as well as an economically important crop (the cotton plant *Gossypium hirsutum*) for amplicon sequencing targeting both bacterial 16S rRNA gene and fungal ITS region, in order to compare the microbial communities under different preservation methods. For primer pairs comparisons, we used the 341F-805R and 799F-1193R primers on the same soil samples.

## Material and Methods

### Plant Preservation Approaches

Plant leaves from *A. caespitosa*, *M. sativa*, *T. triandra* and *C. gayana*, were collected from Pastures And Climate Extremes (PACE) Facility, Western Sydney University, Richmond, Australia. Briefly, each plant species was collected from control monoculture blocks by cutting the leaves with a sterilised scissors before being aseptically transferred into a clear zip lock bag. For the snap freezing and ice incubation methods, plant leaves from each plant species were subsampled into a clean zip lock bag (n = 6 for each treatment) before immediately being stored in liquid nitrogen and on ice, respectively. For air dry method, plant leaf from each plant species were subsampled into a paper bag (n = 6) before being stored in a desiccator filled with silica gel at bottom. Samples were incubated for approximately two days at room temperature until complete dehydration.

Plant leaves, stems and roots from cotton (*G. hirsutum*, genotype Sicot 71BRF) were collected from two-week old cotton plants (10–15 cm tall) grown in a glasshouse with daytime temperature of 32°C and night-time temperature of 25°C. Cotton leaves (top two leaves) and stems (0–5 cm above soil surface) were cut with a sterilised scissors before being transferred into clear zip lock bags (n = 6) while cotton roots were cut and simply washed with distilled water before transferred into a clear zip lock bag. Preservation treatments were conducted as described above.

A total number of 126 frozen plant tissues (~15 mg dry weight, finely cut into ~2 mm × 2 mm pieces) were weighed and DNA was extracted using DNeasy PowerSoil Pro Kit (Qiagen, Hilden, Germany), following the manufacturer’s instructions. Extracted DNA was quality checked by NanoDrop 2000 (Thermo Fisher Scientific, Waltham, Massachusetts, US), quantity checked by Qubit Fluorometer (Thermo Fisher Scientific) and PCR checked to confirm the amplifiability.

Amplicons using 799F/1193R targeting 16S rRNA gene targeting V5-V7 region for bacterial communities to reduce chloroplast sequences from the plant tissue (Chelius and Triplett, 2001), and ITS2 region (FITS7-ITS4R, Ihrmark et al., 2012) for fungal communities were obtained *via* PCR.

### Plant-Based Primer Set Applicability to Soil Samples

We used 86 soil samples from to compare how different primers affect the community determination. These samples were obtained from a glass-house experiment that aimed to evaluate how different verities of rice, soil types, and salinity impact soil microbiomes (unpublished data). DNA for soil samples was extracted after plant harvest as explained above for plants (250 mg of soil was used in the extraction). Each soil sample was collected in a cryogenic tube from the greenhouse and kept under −80°C before DNA extraction. Primer pairs 341F-805R (Herlemann et al., 2011) and 799F-1193R (Chelius and Triplett, 2001) were used to amplify the 16S rRNA gene from all soil samples. Two PCR were performed as: initial denaturation at 95°C for 3 min, followed by 25 cycles consisting of denaturation (95°C for 30 s), annealing (95°C for 30 s) and extension (72°C for 30 s) and a final extension at 72°C for 5 min before merging the samples for downstream process. All sequencing (plant and soil) were performed at Western Sydney University Next Generation Sequencing (NGS) facility (Sydney, Australia) using Illumina MiSeq 2 × 300 bp paired end chemistry. A mock community consists strains belong to order Bacillales, Lactobacillales, Enterobacteriales Pseudomonadales in the following proportion: 47, 28.3, 20.5, and 4.2%, was sequenced with both primers to evaluate the validity of primer comparison. All raw sequence data related to this study are available in the European Nucleotide Archive (The European Bioinformatics Institute, EMBL-EBI) database (Accession No. PRJEB38041).

### Microbial Community Analysis

Raw data obtained from NGS facility were processed using Mothur standard operating procedure (Schloss et al., 2009). Briefly, forward and reverse sequences were merged into contigs. Sequences that contained unidentified bases or had greater than eight homopolymers were filtered out. For bacterial sequences, an additional step aligning sequences against Silva 16S rRNA gene database version 132 (Pruesse et al., 2007) was applied, and unaligned sequences were removed. Refined sequences were pre-clustered (diffs = 1) and chimera checked using UCHIME (Edgar et al., 2011) and singleton was removed to reduce error (Reeder and Knight, 2009). Bacterial and fungal sequences were then taxonomically classified according to the Silva database version 132 and UNITE database version 8, respectively, with 60% cut-off confidence and sequences that match cotton mitochondria, chloroplast, archaea (bacteria) and host ITS regions (fungi) were removed. Remaining sequences were clustered into Operational Taxonomic Units (OTUs) at 100% identity where taxonomy was assigned to, generating 80,617 and 25,577 bacterial and fungal OTUs, respectively.

For plant preservation approaches, the OTU matrices were rarefied to 808 bacterial and 6736 fungal sequences per sample, respectively (**Figures S2A, B**). Rare OTUs (contributed less than 0.1% of total abundances) were removed from the OTU matrices, resulted in 8,218 bacterial OTUs and 3,255 fungal OTUs for downstream analyses. Datasets were analysed using permutational multivariate analysis of variance (Anderson, 2001a) in PRIMER v. 6 (PRIMER-E, UK) to compare bacterial and fungal communities under different preservation methods (snap frozen, ice incubation and air dry). Block effects driving microbial difference were not considered because we only compare difference between preservation treatments. Similarity matrices were calculated based on Bray-Curtis distances on square-root transformed abundance data to compare the composition and abundances of community structure, and on Jaccard distances to compare the presence/absence of the community members in PRIMER. Analyses used 9,999 permutations of residuals under a reduced model (Anderson, 2001b). Pair-wise analyses were performed to compare the differences between preservation methods, and p-values were adjusted following Holm’s method (Holm, 1979) to reduce the bias generated in statistical analysis. Permutational multivariate dispersion (PERMDISP) analysis was used to test for homogeneity of multivariate dispersion within groups in PRIMER (Anderson, 2006). Alpha and beta diversity were analysed using R package “phyloseq”. Data visualisation including Principal Coordinates Analysis (PCoA) plots were generated based on Bray–Curtis and Jaccard distance, and taxonomic analysis based on the Bray–Curtis dissimilarity matrix with heatmap were performed using R packages “phyloseq”, “dplyr” and “ggplot2” (Lozupone et al., 2012).

To identify the influence of preservation methods on dominant and rare microbial taxa, we followed the definition from Soliveres et al. (2016) to extract dominant communities (the top 10% of OTUs in terms of abundance) and rare communities (the bottom 10% OTUs) from the OTU tables generated with Mothur, respectively. PERMANOVA, alpha and beta diversity analyses were applied following the methods described above.

To compare the two datasets using different primer pairs on soil bacterial communities, both datasets using two sets of primers (341F/805R and 799F/1193R) with 12,199 and 16,229 bacterial raw OTUs, respectively, were rarefied to 8,000 sequences per sample (**Figures S2C, D**) with 9,317 and 13,737 OTUs, respectively. Alpha diversity analysis and correlation between two datasets, as well as mantel test based on Bray–Curtis measures estimating the beta diversity correlation between two datasets were conducted in R. Microbial composition was also analysed with R package “phyloseq”.

## Results

### Effect of Preservation Methods on Plant Microbiomes

**Figure 1:**
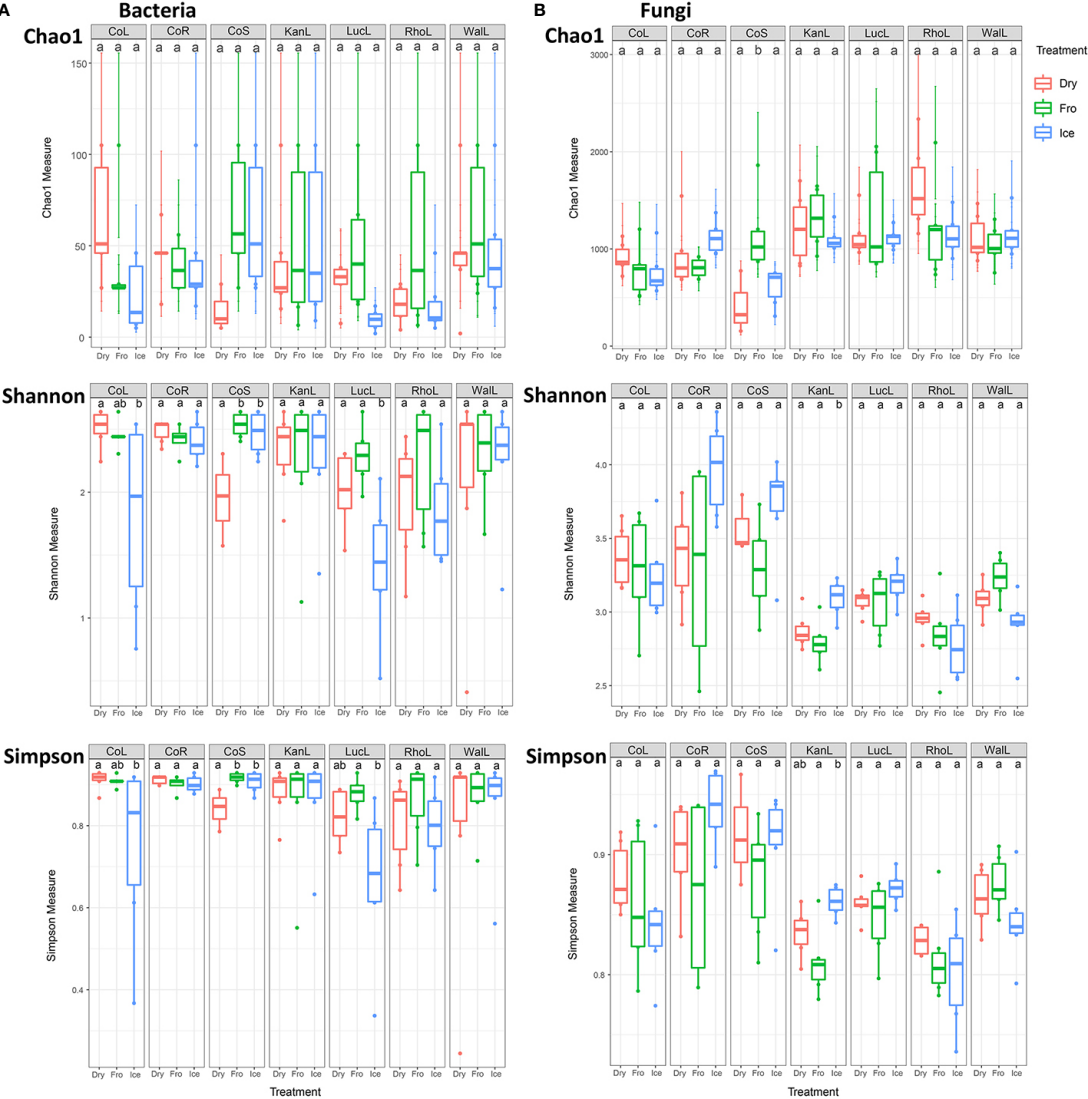
Alpha diversity (Chao1, Shannon and Simpson) indices of bacterial **(A)** and fungal **(B)** communities under different preservation methods. Dry = air-dry (red), Fro = snap freezing (green), Ice = ice incubation (blue). CoL = cotton leaf, CoR = cotton root, CoS = cotton stem, KanL = kangaroo leaf, LucL = lucerne leaf, RhoL = rhodes leaf, WalL = wallaby leaf.

**Figure 2:**
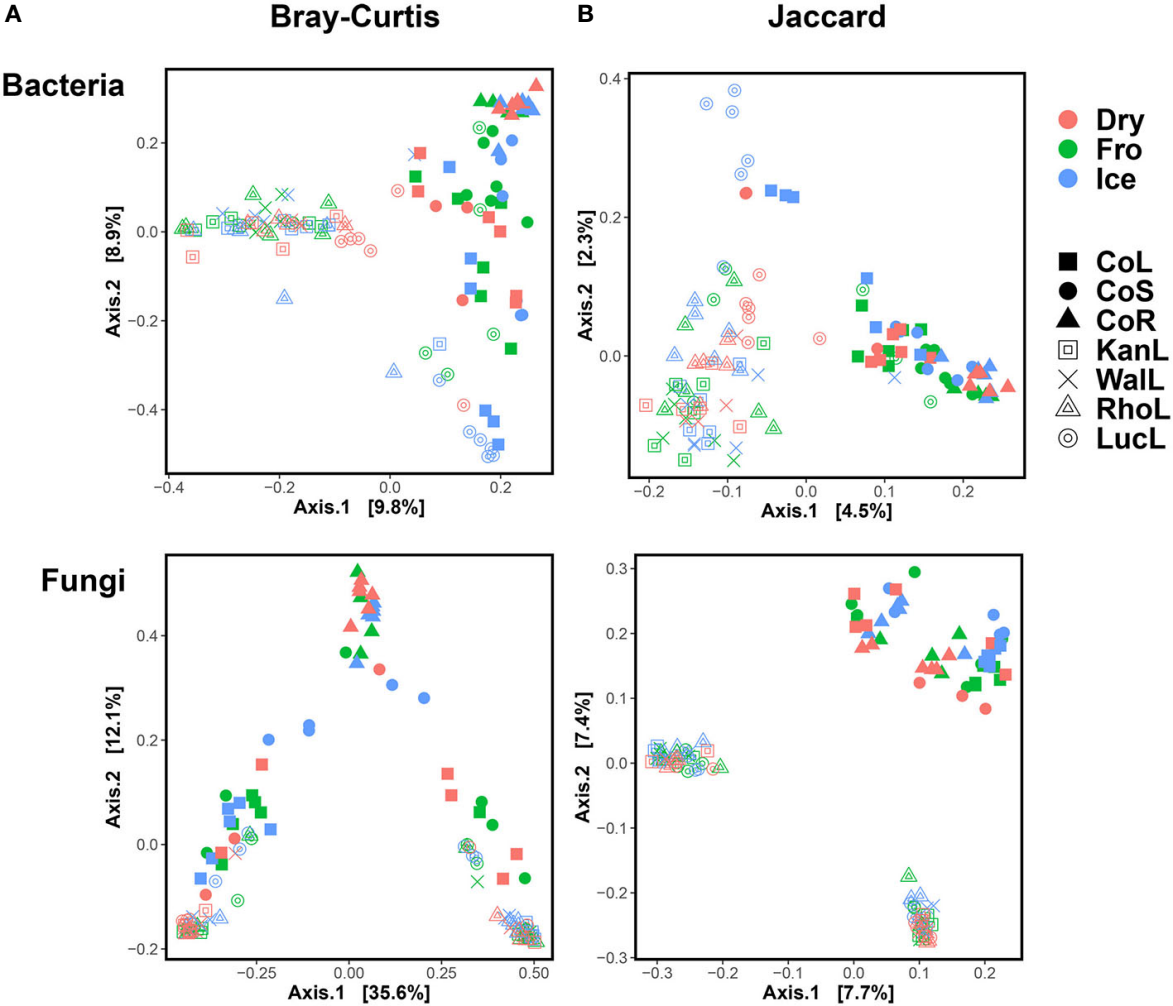
Principal Coordinates Analysis (PCoA) plot using Bray–Curtis and Jaccard distance matrix on bacterial **(A)** and fungal **(B)** communities under different preservation methods. Dry = air-dry (red), Fro = snap freezing (green), Ice = ice incubation (blue). CoL = cotton leaf (solid square), CoS = cotton stem (solid circle), CoR = cotton root (solid triangle), KanL = kangaroo leaf (open square), WalL = wallaby leaf (cross), RhoL = rhodes leaf (open triangle), LucL = lucerne leaf (open circle).

**Figure 3:**
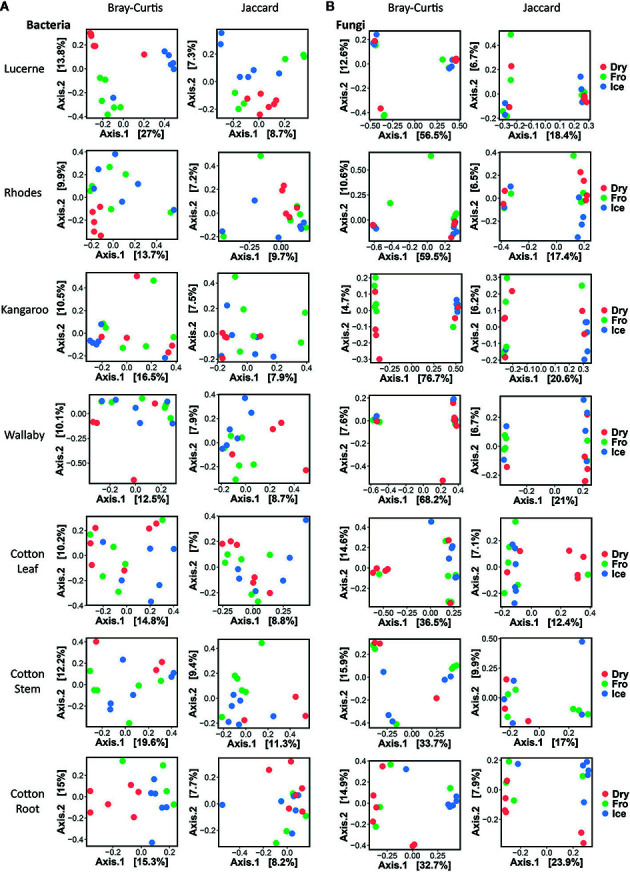
Individual Principal Coordinates Analysis (PCoA) plot using Bray–Curtis and Jaccard distance matrix on bacterial **(A)** and fungal **(B)** communities under different preservation methods.

**Figure 4:**
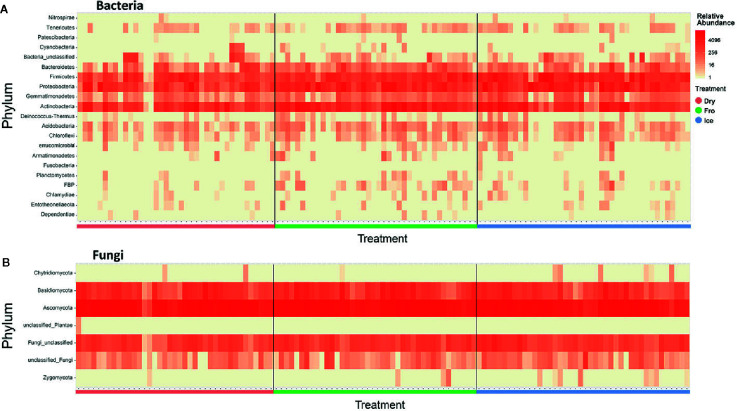
Heatmap indicating relative abundance of bacterial **(A)** and fungal **(B)** phyla across all samples. Dry = air-dry (red), Fro = snap freezing (green), Ice = ice incubation (blue).

In leaf preservation approach, a total number of 8,218 bacterial and 3,215 fungal OTUs were analysed in the preservation experiment. Five plant DNA samples were dropped due to low DNA quality and poor sequencing reads, which end up with 121 samples in total. Generally, there were no significant difference of species richness and evenness among the different preservation treatment observed from the alpha indices (Chao1, Shannon and Simpson, **Figure 1**; P >0.05) with a few exceptions due to low diversities (In bacterial communities, Shannon index – Dry ≠ Ice in cotton leaf, Dry ≠ Fro = Ice in cotton stem, Dry = Fro ≠ Ice in lucerne leaf; Simpson index – Dry ≠ Ice in cotton leaf, Dry ≠ Fro = Ice in cotton stem, Fro ≠ Ice in lucerne leaf. In fungal communities, Chao1 index – Fro ≠ Ice = Dry in cotton stem; Shannon index – Dry = Fro ≠ Ice in kangaroo leaf; Simpson index – Fro ≠ Ice in kangaroo leaf, P <0.05). When comparing the bacterial structure (Bray–Curtis dissimilarity) between sample groups and preservation treatments from PERMANOVA tests (**Table 1**), no significant differences (P >0.05) were found between preservation treatments except for lucerne leaves wherein we found some small differences for ice incubation and frozen, ice incubation and air-dry treatments (P <0.05), respectively, and for cotton roots between ice incubation and air dry treatments (P <0.05). PERMDISP tests indicated that differences in lucerne leaves (F = 5.128, df1 = 2, df2 = 15, P = 0.058) and cotton roots (F = 1.167, df1 = 2, df2 = 13, P = 0.643) were likely driven by preservation methods. For bacterial identity (presence/absence, Jaccard dissimilarity), no significant difference was found between preservation treatments (**Table 1A**). PCoA plots showed difference of bacterial abundances and identities assembled on different plant species and tissue (**Figure 2A**), but differences between preservation methods within each plant species and tissue were found matching the PERMANOVA test (**Figure 3A**). In the subset of dominant bacterial communities, no significant difference was found in Bray–Curtis dissimilarity except in lucerne leaves between ice incubation and air-dry preservation methods, and no significant difference was found across all samples in Jaccard dissimilarity (**Table 2A**). In the subset of rare microbial communities, no significant difference was found across all samples in either Bray–Curtis or Jaccard dissimilarities (**Table 2A**). In bacterial structure and composition, no clear patterns can be found between preservation methods at the phylum level (**Figure 4A**).

**Table 1:** Pairwise PERMANOVA analyses of bacterial (A) and fungal (B) communities based on Bray–Curtis and Jaccard measures of square-root transformed relative abundances of plant bacterial communities under different treatments (snap frozen, ice incubation and air dry)..

	(A) Bacterial Community		(B) Fungal Community	
	Bray-Curtis	Jaccard	Bray-Curtis	Jaccard
Kangaroo Leaf	NSD	NSD	NSD	NSD
Rhodes Leaf	NSD	NSD	NSD	NSD
Wallaby Leaf	NSD	NSD	NSD	NSD
Lucerne Leaf	**Fro ≠ Ice, Ice ≠ Dry**	NSD	NSD	NSD
Cotton Leaf	NSD	NSD	NSD	NSD
Cotton Stem	NSD	NSD	NSD	NSD
Cotton Root	**Ice ≠ Dry**	NSD	NSD	NSD

NSD, no significant difference Significant results (P < 0.05) highlighted with bold.

**Table 2:** Pairwise PERMANOVA analyses of dominant and rare bacterial (A) and fungal (B) communities based on Bray–Curtis and Jaccard measures of square-root transformed relative abundances of plant bacterial communities under different treatments (snap frozen, ice incubation and air dry).

	(A) Bacterial Community				(B) Fungal Community			
	Dom.bray	Dom.jac	Rare.bray	Rare.jac	Dom.bray	Dom.jac	Rare.bray	Rare.jac
Kangaroo Leaf	NSD	NSD	NSD	NSD	NSD	NSD	NSD	NSD
Rhodes Leaf	NSD	NSD	NSD	NSD	NSD	NSD	NSD	NSD
Wallaby Leaf	NSD	NSD	NSD	NSD	NSD	NSD	NSD	NSD
Lucerne Leaf	**Ice ≠ Dry**	NSD	NSD	NSD	NSD	NSD	NSD	NSD
Cotton Leaf	NSD	NSD	NSD	NSD	NSD	NSD	NSD	NSD
Cotton Stem	NSD	NSD	NSD	NSD	NSD	NSD	NSD	NSD
Cotton Root	NSD	NSD	NSD	NSD	NSD	NSD	NSD	NSD

Dom, Dominant; Bray, Bray–Curtis; Jac, Jaccard; NSD, no significant difference. Significant results (P < 0.05) highlighted with bold.

In fungal community, no significant difference was found in either fungal community structure based on Bray–Curtis dissimilarity or composition based on Jaccard dissimilarity (**Table 1B**). PCoA plots showed similar fungal abundances and identities across all plant species except cotton (**Figure 2B**), but differences between preservation methods within each plant species and tissue were found matching the PERMANOVA test (**Figure 3B**). Regardless of the plant species and tissue, different preservation methods did not influence the microbial communities. In the subset of dominant and rare fungal communities, no significant difference was found in either Bray–Curtis or Jaccard dissimilarities across all dominant and rare fungal communities (**Table 2B**). In fungal structure and composition, no clear pattern can be found between preservation methods under phylum level (**Figure 4B**).

### Assessing the Utility of Plant-Based Bacterial Primer Pairs for Soil Samples

**Figure 5:**
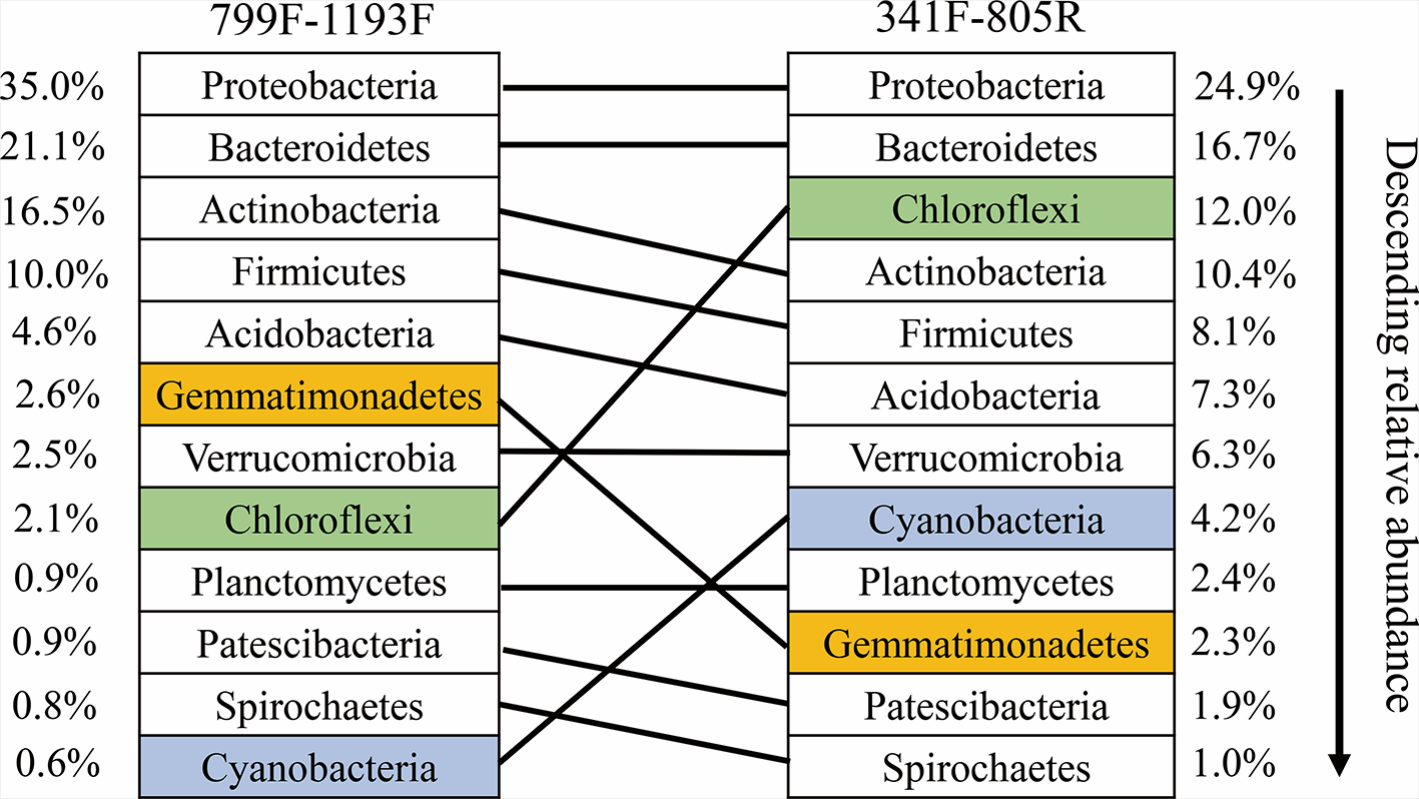
Ranked relative abundant of the top-12 dominant bacterial phyla as determined by the primer pairs 799F/1193F and 341F/805R, respectively. The same phylum amplified by the two primer pairs are linked by line.

**Figure 6:**
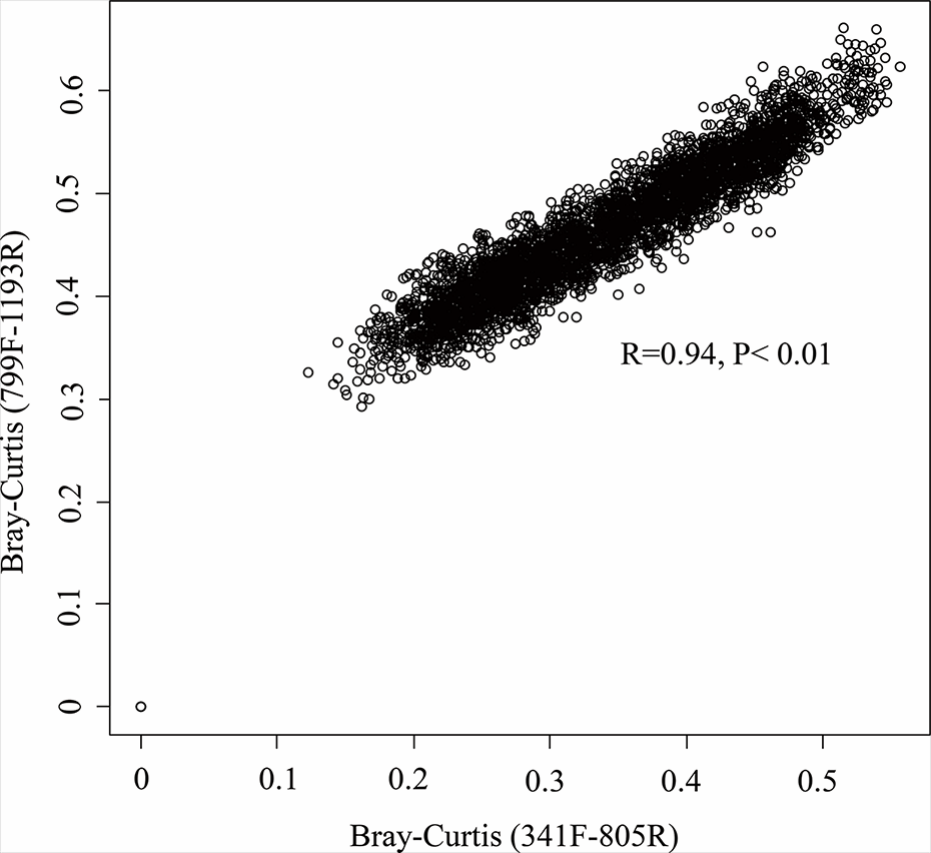
Relationship between the community composition as determined by the primer pairs 799F/1193F and 341F/805R. Mantel correlation was performed on the Bray–Curtis matrix at the OTU level.

Our results indicate that both primer sets provide similar results, and that overall community compositional data from the plant-based primer set (799F/1193R) were directly comparable to that obtained from the 341F/805R primer set (soil primer sets). In general, the 799F/1193R primer generated higher alpha diversity than 341F/805R (**Table 3**), but the variation trends were similar (**Figure S1**). All the diversity metrics, including Shannon diversity, richness (Chao1) and Faith’s phylogenetic diversity were highly correlated between the two primer sets (**Figure S1**). The two primer pairs generated the same abundant phylum (top 12), and these phyla accounted for 97.5% of the total bacterial abundance in both datasets (**Figure 5**). The ranking order of the abundant phylum were similar for both primer pairs except for two bacterial phyla (Chloroflexi and Cyanobacteria) with photosynthesis abilities. At the OTU level, the community compositions were highly correlated between the two datasets, and different primer pairs did not affect the treatment effect on bacterial community composition (**Figure 6**). In the result of mock community amplification, primer pair 341F/805R resulted in 51.5% Bacillales, 19.4% Lactobacillales, 24.7% Enterobacteriales and 4.4% Pseudomonadales, while primer pair 799F/1193R resulted in 42.5% Bacillales, 25.1% Lactobacillales, 25.6% Enterobacteriales and 6.8% Pseudomonadales (**Figure S3**). The result of bacterial community using two primer sets showed similar proportions compare to standard mock community, indicating the validity of the result.

**Table 3:** Alpha diversity measure (Mean ± SD) of soil bacterial communities using two different primer pairs.

Primer pair	Shannon	Faith’s PD	Chao1
341F/805R	9.03 ± 0.41	121.2 ± 15.3	2357.0 ± 300.4
799F/1193R	9.66 ± 0.40	136.6 ± 14.4	4162.5 ± 377.5

## Discussion

Our study provides strong evidence that preservation strategies have minor, if any impact on the plant leaves, stems and roots microbiomes for multiple plant species belonging to contrasting functional groups (e.g. C3, C4, N-Fixers, etc.). Moreover, we found that plant and soil microbiomes might be directly comparable in future studies as widely used plant-based primer set (799F/1193R) produced similar results to those from the primer set used widely in soil microbiome studies (341F/805R) in terms of diversity and community composition across contrasting soil types. These findings imply that multiple approaches are available to accommodate different research logistics and needs without compromising the reliability of findings. This information is critical to overcome some of the critical logistics challenges associated with large-scale studies on the plant microbiome at the regional and global scales, and also indicate that amplicon sequencing for bacterial communities are robust to primer set bias.

### Contrasting Preservation Methods Do Not Alter the Plant Microbiome Structure

Microbiomes associated with plant tissue are variable and could be impacted by multiple factors such as environmental changes, plant-microbe interactions and microbe-microbe interactions (Singh and Trivedi, 2017; Hamonts et al., 2018). Major concerns with sample preservation are mainly associated with increased in temperature (above −20°C) because of the leaf disintegration under high temperature, commonly reported in leaf litter (Dilly et al., 2001; Voříšková and Baldrian, 2013; Shay, 2016). The preservation methods implemented in this study tested a range of preservation temperatures, which overall did not affect the bacterial and fungal communities (except for bacterial community of lucerne leaf and cotton root) associated with different parts of plants, suggesting reliable data could be obtained from all preservation methods used in this study.

In addition to the overall communities, we also investigated the dominant and rare communities separately to avoid omissions of potential microbial variation from less abundant microbes, given the potential functional role of microbial communities in the ecosystem (Nazaries et al., 2013; Soliveres et al., 2016). Consistent with the overall community patterns, in dominant communities remain unchanged except for bacterial structure of lucerne leaves (P <0.05, **Table S1B**). In contrast, in rare communities, no significant difference was found between preservation methods across all plant species and tissue. Collectively, the small changes in microbiome of lucerne leaves were present only in dominant bacterial communities but not rare communities, indicating that difference observed was mainly driven by shifts in the most abundant species rather than the rare ones.

In microbial composition, variation could be observed between samples, but no significant pattern was found between preservation methods. This result was consistent for both bacterial and fungal communities (**Figure 4**). Our findings are supported from those of other similar studies on different biological materials such as feces, soil and insects, which also reported little effect of temperature, storage method and duration on microbial communities (Lauber et al., 2010; Dominianni et al., 2014; Hammer et al., 2015). Therefore, the preservation methods in this study have provided a new perspective to overcome the difficulties of bulk sampling in regional or remote areas.

### Plant-Based Primer Sets Are Comparable to Those From Soil Surveys

Primer selection is one of the key factors in microbiome analyses. Primer pairs 341F/805R and 515F/806R are widely accepted for bacterial community analysis from human, insects, soil, plant and marine species (Caporaso et al., 2011; Caporaso et al., 2012; Jakobsson et al., 2014; Delgado-Baquerizo et al., 2016; Walters et al., 2016; Gomez-Polo et al., 2017; Hamonts et al., 2018; Clerissi et al., 2020), while primer pair 799F/1193R has a two-base pair mismatch for chloroplast (Chelius and Triplett, 2001), which is more suitable for plant microbiome analysis.

However, plant microbiome studies usually require both soil and plant microbiome profiles to connect underground and aboveground microbial communities (Liu et al., 2017; Hamonts et al., 2018), which essentially need consistency with primer selections. Therefore, to priorly remove the contamination of chloroplast from plant tissue, primer pair 799F/1193R is preferred in the plant microbiome analyses.

In our study, the two primer pairs showed similar patterns of relative abundance and composition of bacterial communities the soil samples (**Figures 5** and **6**). A lower Cyanobacteria abundance were found in the bacterial community using primer pair 799F/1193R because of the chloroplast mismatch, which was evidenced in previous studies (Beckers et al., 2016; Thijs et al., 2017). Despite the minor variation of a few bacterial phyla between the communities using two different primer sets, the overall bacterial structures were highly correlated (R^2^ = 0.94, P < 0.01, **Figure 6**), which enhanced the previous finding of higher coverage using primers targeting V3–V4 and V5–V7 hypervariable regions (Thijs et al., 2017). This result suggests that datasets using these two primer pairs on microbiome studies are comparable, and primer pair 799F/1193R use for soil microbiome studies is valid.

## Conclusion

In this study, we 1) sampled multiple plant species with different functional pathways and leaf architectures to identify the impact of different preservation method on plant microbiome, and 2) evaluated the validity using plant specific primer pair 799F/1193R on soil microbiome approach. The preservation methods used in this study did not impact either the bacterial community or the fungal community, and this pattern was consistent across most of the plant species. While more robust preservation methods to be implemented in the future is possible, the result from this study could significantly help large-scale sampling at regional and global scales, particularly in remote areas, with air-dry or ice incubation method.

The two different pairs of primers on bacterial plant microbiome analysis resulted in similar bacterial abundance and composition, indicating that the mismatch primer pair 799F/1193R designed for plant microbiome analysis, could also be used on other non-plant samples when Cyanobacteria was not considered. Our result facilitated the sampling on global-scaled plant microbiome studies and enables researchers to perform combined soil and plant microbiome analyses.

## Data Availability Statement

All raw sequence data related to this study are available in the European Nucleotide Archive (The European Bioinformatics Institute, EMBL-EBI) database (Accession No. PRJEB38041).

## Author Contributions

ZQ, JW, MD-B, PT, EE, and BS conceived and designed the study. ZQ, JW, Y-MC, and HZ collected the samples. ZQ, Y-MC, and JW processed the samples and data analyses. ZQ wrote the first draft of the manuscript which was revised by all co-authors. MD-B, PT, and EE revised the manuscript. All authors contributed to the article and approved the submitted version.

## Funding

This work is funded by Australian Research Council (DP170104634) grant.

## Conflict of Interest

The authors declare that the research was conducted in the absence of any commercial or financial relationships that could be construed as a potential conflict of interest.
