# Supplementary material for: Plant Microbiomes: Do Different Preservation Approaches and Primer Sets Alter Our Capacity to Assess Microbial Diversity and Community Composition?
Source: Front Plant Sci. 2020 Jul 3;11:993. doi: 10.3389/fpls.2020.00993 (PMC7351510; doi:10.3389/fpls.2020.00993)
Supplement: Supplementary file 1 [file DataSheet_1.zip › New folder (2)/Table S1 and Figure captions.docx]

**Supplementary Materials**

**Supplementary Table 1.** Adjusted p-value of pairwise test in (A) overall communities, (B) dominant communities and (C) rare communities. Kan = Kangaroo, Wal = Wallaby, Rho = Rhodes, Luc = Lucerne, CoL = Cotton Leaf, CoS = Cotton Stem, CoR = Cotton Root. Fro = snap freezing, Ice = ice incubation, Dry = air dry. Significant results (P < 0.05) highlighted with bold.

|  | 1. **Overall** | | | | 1. **Dominant** | | | | 1. **Rare** | | | |
| --- | --- | --- | --- | --- | --- | --- | --- | --- | --- | --- | --- | --- |
| Pairwise test | 16S.Bray | 16S.Jaccard | ITS.Bray | ITS.Jaccard | 16S.Bray | 16S.Jaccard | ITS.Bray | ITS.Jaccard | 16S.Bray | 16S.Jaccard | ITS.Bray | ITS.Jaccard |
| Kan_Fro_Ice | 0.219 | 0.905 | 0.13 | 0.116 | 0.297 | 0.406 | 0.063 | 0.32 | 0.945 | 1 | 0.147 | 0.105 |
| Kan_Fro_Dry | 0.801 | 0.845 | 1 | 1 | 0.720 | 0.808 | 1 | 1 | 0.845 | 1 | 1 | 1 |
| Kan_Ice_Dry | 0.522 | 0.905 | 0.735 | 0.509 | 0.511 | 0.808 | 0.91 | 0.512 | 0.900 | 1 | 0.624 | 0.864 |
| Wal_Fro_Ice | 0.522 | 0.140 | 1 | 1 | 0.511 | 0.756 | 1 | 1 | 0.608 | 0.238 | 1 | 1 |
| Wal_Fro_Dry | 0.094 | 0.212 | 1 | 1 | 0.128 | 0.190 | 0.975 | 1 | 0.190 | 0.234 | 1 | 1 |
| Wal_Ice_Dry | 0.159 | 0.141 | 1 | 1 | 0.297 | 0.256 | 1 | 1 | 0.189 | 0.147 | 1 | 1 |
| Rho_Fro_Ice | 0.801 | 0.905 | 1 | 1 | 0.720 | 0.808 | 1 | 1 | 0.945 | 1 | 1 | 1 |
| Rho_Fro_Dry | 0.080 | 0.905 | 1 | 1 | 0.072 | 0.808 | 1 | 1 | 0.845 | 1 | 1 | 1 |
| Rho_Ice_Dry | 0.427 | 0.905 | 1 | 1 | 0.297 | 0.808 | 1 | 1 | 0.945 | 1 | 1 | 1 |
| Luc_Fro_Ice | **0.044** | 0.630 | 1 | 1 | 0.060 | 0.285 | 1 | 1 | 0.945 | 1 | 1 | 1 |
| Luc_Fro_Dry | 0.058 | 0.845 | 1 | 1 | 0.102 | 0.808 | 1 | 1 | 0.945 | 1 | 1 | 1 |
| Luc_Ice_Dry | **0.040** | 0.101 | 1 | 1 | **0.021** | 0.084 | 1 | 1 | 0.493 | 0.432 | 1 | 1 |
| CoL_Fro_Ice | 0.518 | 0.845 | 0.456 | 1 | 0.511 | 0.765 | 0.306 | 1 | 0.945 | 1 | 1 | 1 |
| CoL_Fro_Dry | 0.522 | 0.845 | 1 | 1 | 0.496 | 0.760 | 1 | 1 | 0.945 | 1 | 1 | 1 |
| CoL_Ice_Dry | 0.146 | 0.255 | 0.132 | 0.154 | 0.128 | 0.234 | 0.2 | 0.084 | 0.720 | 0.51 | 0.437 | 0.396 |
| CoS_Fro_Ice | 0.641 | 0.845 | 1 | 1 | 0.720 | 0.808 | 1 | 1 | 0.756 | 1 | 1 | 1 |
| CoS_Fro_Dry | 0.159 | 0.212 | 0.698 | 0.389 | 0.180 | 0.255 | 0.688 | 0.432 | 0.252 | 0.228 | 0.468 | 0.544 |
| CoS_Ice_Dry | 0.159 | 0.212 | 0.391 | 0.415 | 0.130 | 0.140 | 0.459 | 0.442 | 0.189 | 0.180 | 0.420 | 0.280 |
| CoR_Fro_Ice | 0.801 | 0.905 | 0.698 | 1 | 0.720 | 0.808 | 0.688 | 1 | 0.945 | 1 | 1 | 1 |
| CoR_Fro_Dry | 0.146 | 0.487 | 1 | 1 | 0.128 | 0.756 | 1 | 1 | 0.845 | 0.644 | 1 | 1 |
| CoR_Ice_Dry | **0.049** | 0.563 | 0.384 | 0.365 | 0.060 | 0.702 | 0.266 | 0.323 | 0.945 | 1 | 0.468 | 0.361 |

**Figure S1:** Relationship between the different diversity metrics from the primer pairs 799F/1193F (Y) and 341F/805R (X). Correlation was determined using two-tailed Pearson.

**Figure S2:** Rarefaction curve for the sequences of plant microbiomes obtained from (A) bacterial 16S rRNA gene sequencing and (B) fungal ITS region sequencing.

**Figure S3:** Microbial composition of mock communities using two primer sets (341F/805R in blue, 799F/1193R in orange and standard proportion of mock community in red).
