# Supplementary figures and images for: Plant Microbiomes: Do Different Preservation Approaches and Primer Sets Alter Our Capacity to Assess Microbial Diversity and Community Composition?
Source: Front Plant Sci. 2020 Jul 3;11:993. doi: 10.3389/fpls.2020.00993 (PMC7351510; doi:10.3389/fpls.2020.00993)

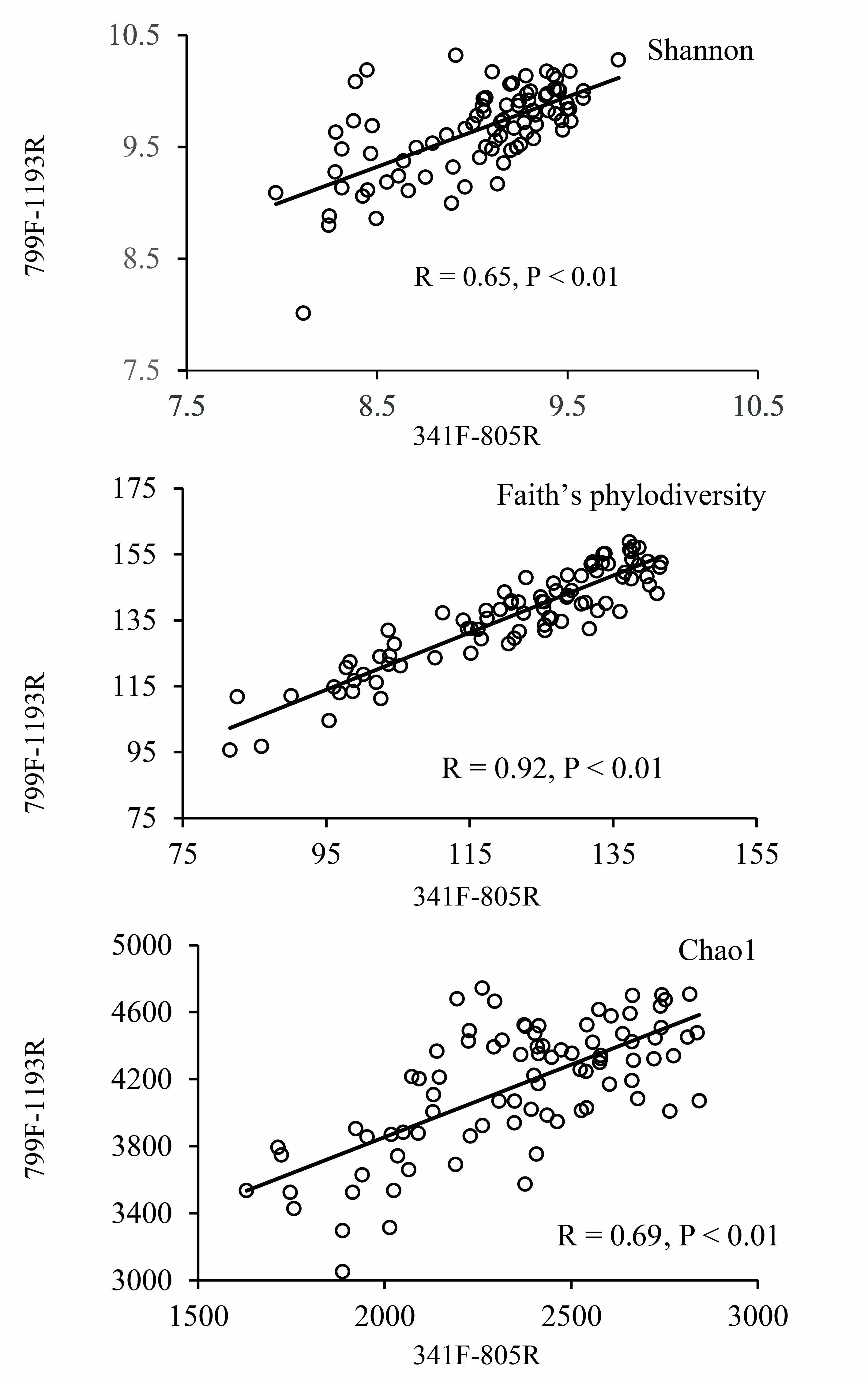

Supplement: Supplementary file 2 [file Image_1.tiff]

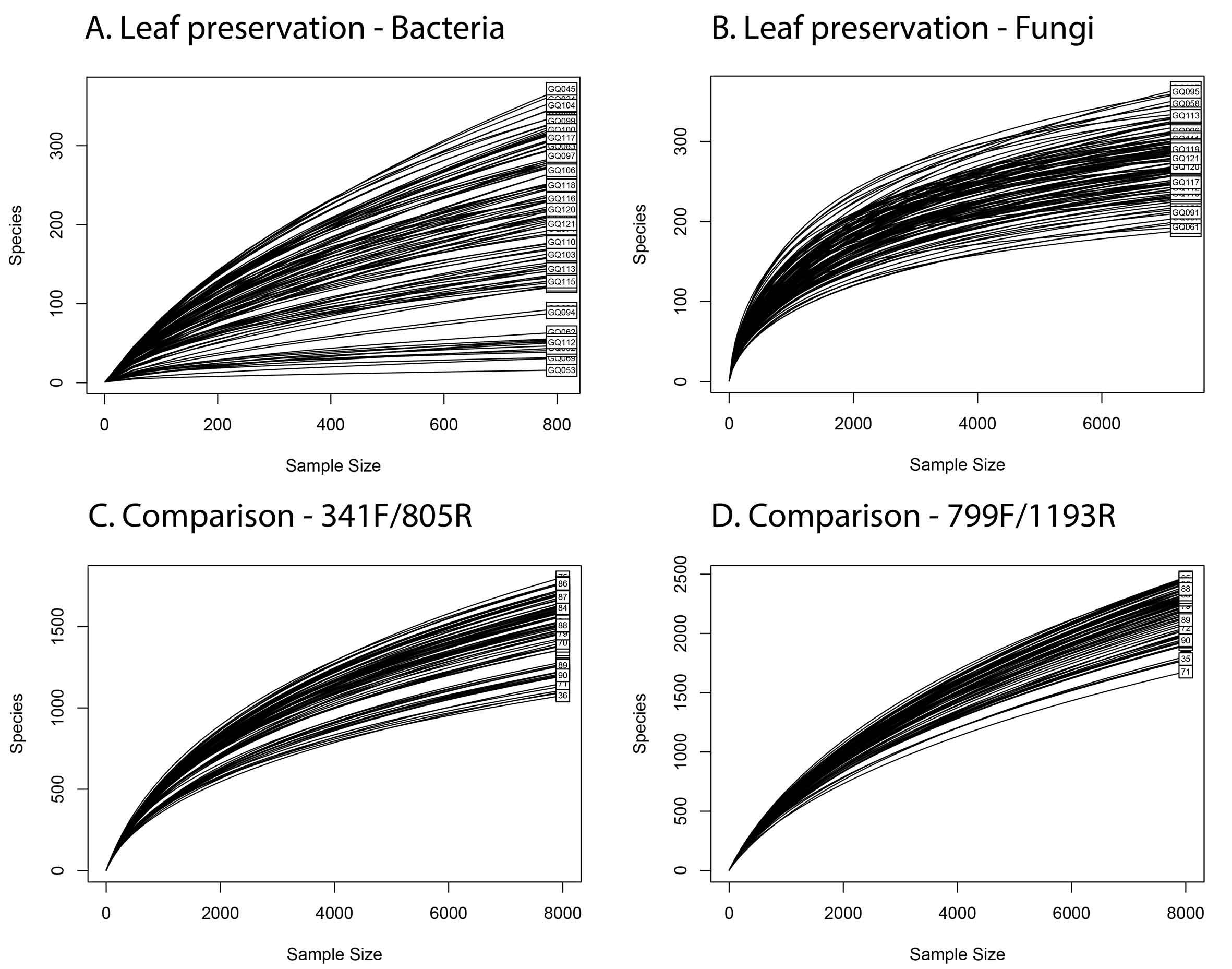

Supplement: Supplementary file 3 [file Image_2.jpeg]

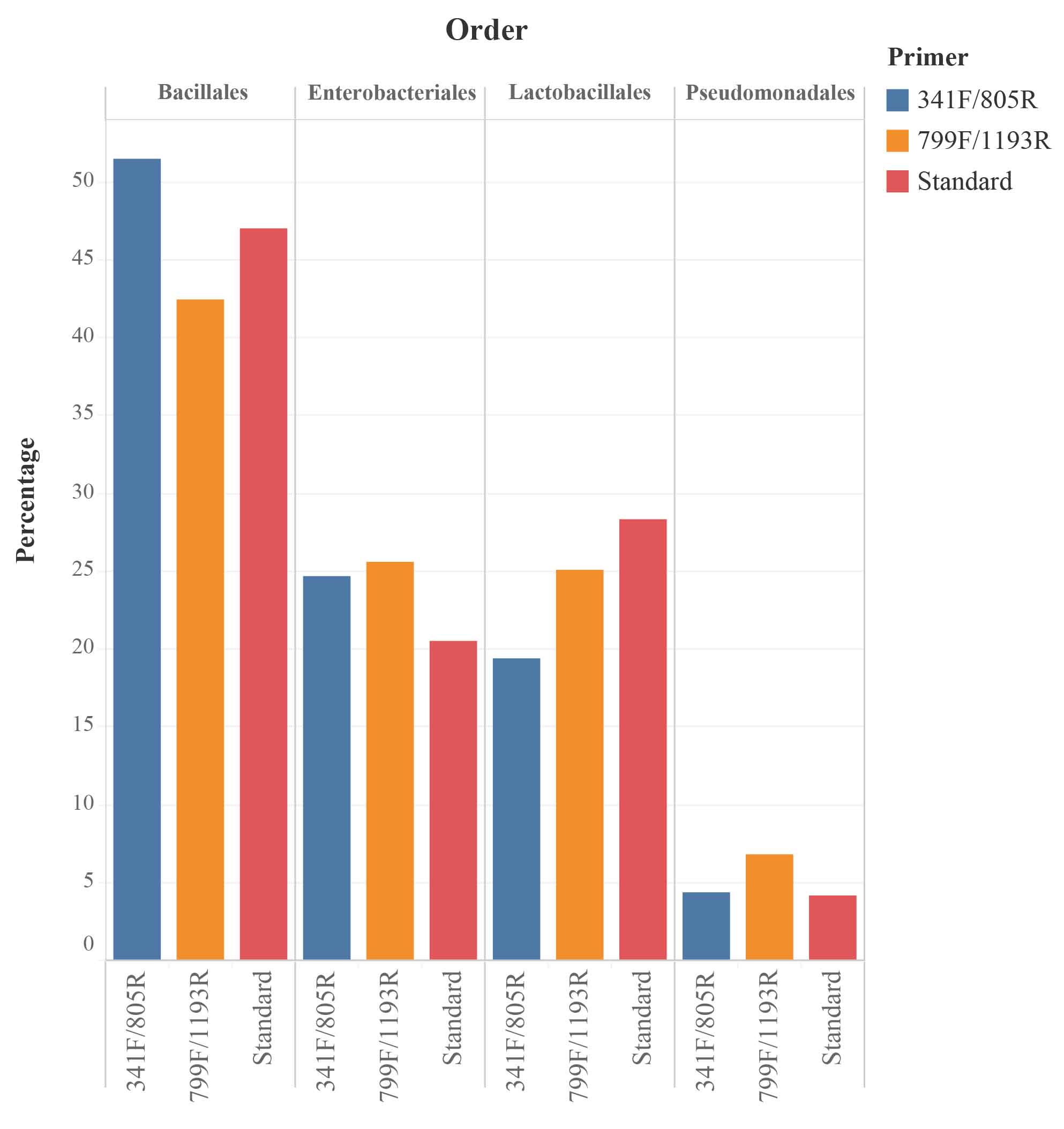

Supplement: Supplementary file 4 [file Image_3.jpeg]
